# Application of magnetic nanoparticles in cell therapy

**DOI:** 10.1186/s13287-022-02808-0

**Published:** 2022-04-01

**Authors:** Yuling Chen, Shike Hou

**Affiliations:** 1 Institute of Disaster and Emergency Medicine, Tianjin University, Tianjin, China; 2 Tianjin Key Laboratory of Disaster Medicine Technology, Tianjin, China

**Keywords:** MNPs, Cell therapy, Magnetic targeting, Drug delivery

## Abstract

Fe_3_O_4_ magnetic nanoparticles (MNPs) are biomedical materials that have been approved by the FDA. To date, MNPs have been developed rapidly in nanomedicine and are of great significance. Stem cells and secretory vesicles can be used for tissue regeneration and repair. In cell therapy, MNPs which interact with external magnetic field are introduced to achieve the purpose of cell directional enrichment, while MRI to monitor cell distribution and drug delivery. This paper reviews the size optimization, response in external magnetic field and biomedical application of MNPs in cell therapy and provides a comprehensive view.

## Introduction

Stem cells have the function of tissue regeneration and repair. In addition, EVs and exosomes from cells also have the function of repairing injury. In particular, they have the attributes of low immunogenicity and drug delivery potential, and they carry a variety of signaling biomolecules (proteins, mRNAs and miRNAs). At present, stem cells and their secretory vesicles have been widely used in disease treatment. Researchers intravenously inject repair cells or vesicles into organisms to treat diseases, hoping that they can accumulate at the injured site via the blood circulation [1]. The key is the successful delivery of drugs to the lesion. Although stem cells have homing ability, they still have shortcomings. The actual enrichment effect is not ideal [2, 3]. Kyrtatos et al. [4] showed data in vitro by computer simulation that a few cells were retained in the target tissue area in the presence of blood flow. By intracoronary injection, the cell residence time was only a few minutes in the study of myocardial regeneration [5]. At the same time, EVs were cleared in a short time (1.2–1.3 min) after intravenous injection into the blood, and most of them accumulated in the liver and spleen. Therefore, the poor targeting, the low retention rate and the poor therapeutic effect limit the clinical application in cell therapy [6].

SPIONs have obvious advantages of low toxicity to organisms, magnetic targeting, magnetic resonance imaging tracking, hyperthermia and drug delivery ability. More importantly, SPIONs are an iron-containing preparation approved by the FDA. Therefore, the development of SPIONs has attracted increasing attention, especially in medicine. It is important to target and transport the therapeutic cells and EVs under the joint action of MNPs and an external magnetic field. This method can not only improve precise positioning within organisms [4, 7–9], improve the retention rate and prolong the drug half-life but also reduce the drug dosage and enhance the efficacy, even in high-flow systems (such as the arterial vascular system). In the literature [4, 10], it was found that targeted stem cells were enriched approximately five times more than nontargeted cells in arteries. After labeling with MNPs, EVs were preserved in the blood for 7–8 h in a magnetic field. Magnetic targeting was found to enhance myocardial retention of intravascular EPCs. They could also effectively cross the BBB and deliver drugs [11, 12] under a magnetic field. Therefore, cells and EVs labeled by MNPs achieved multiple functions of treatment, targeting, drug delivery, magnetic hyperthermia and MRI, which has a great development potential in medicine.

Here, we introduce the combination of MNPs not only with cells, but also with extracellular vesicles, exosomes and artificial simulated liposomes (known as membrane system or membrane structure for convenience). In this paper, we review the requirements for the size of MNPs when used in combination with membrane systems, the response under external magnetic field and its application in medicine.

## Application size of MNPs

In this paper, we have reviewed the literature from the last 20 years and further explained the importance of the appropriate size of MNPs in Table 1.

**Table 1:** Literature summary of medical applications of MNPs in recent 20 years

	MNP	Core size (nm)	Carrier	Disease	Function	References
1	CoFe_2_O_4_	10	Cell	–	–	[13]
2	Endorem	3–5	Cell	Vascular injury	MT	[4]
3	γ-Fe_2_O_3_	7 & 9	Liposome	Cancer	MT and hyperthermia and MRI	[14]
4	Fe_3_O_4_	10	Liposome	–	MT	[15]
5	Fe_3_O_4_	6.8 ± 1.36	Liposome	Tumor	MT	[16]
6	Fe_3_O_4_	30–40	–	Microglial BV2 cells	Magnetic hyperthermia	[17]
7	Fe_3_O_4_	10	Hydrogel vesicle	–	Drug delivery	[18]
8	γ-Fe_2_O_3_	8	Liposome	Prostatic adenocarcinoma	MT	[19]
9	γ-Fe_2_O_3_	8	Microvesicle	–	MRI and MT	[20]
10	–	19 ± 3	–	Cancer	Magnetic hyperthermia	[11]
11	NPs	5 /10	Liposome	–	Delivery	[12]
12	Fe_3_O_4_	16 ± 4	Liposome	–	MRI	[21]
13	Fe_3_O_4_	200	EV	Myocardial infarction	MT	[22]
14	Fe_3_O_4_	100	Exosome	Wound healing	MT	[23]
15	VivoTrax	–	Exosome	Tumor	MPI	[24]
16	Fe_3_O_4_	∼ 10	Exosome	Cancer	MT	[25]
17	Fe_3_O_4_	∼ 10	Exosome	Tumor	MT and delivery	[26]
18	Fe_3_O_4_	< 60	Exosome	Wound healing	MT	[27]
19	SPION	10	Exosome	Cancer	Delivery	[28]
20	Fe_3_O_4_	8	Exosome	Glycuresis	MT	[29]
21	Fe_3_O_4_	20 & 200	MSC	Trabecular meshwork	MT	[30]
22	SPION	6.2	MSC	Spinal cord injury	MT	[31, 32]
23	ZnFe_2_O_4_	18.93 ± 1.6	MSC	Cancer	Magnetic hyperthermia	[33]
24	Fe_3_O_4_	6–7	MSC	–	MRI	[34]
25	Fe_3_O_4_	8	ESC	–	MT	[35]
26	Fe_3_O_4_	6.6	ADSC	Osteoporosis	MT	[36]
27	Fe_3_O_4_	10	MSC	Angiogenesis	MT	[37]
28	Fe_3_O_4_	10	hMSC	–	MRI and MT	[38]
29	γ-Fe_2_O_3_	1.7–11.5	MSC	Pulmonary damage	MT	[39]
30	SPION	10	ADSC	Parkinson	MT	[40]
31	Zn_0.4_Fe_2.6_O_4_	15	NSC	Brain stroke	MT	[41]
32	IONP	∼ 22	H9C2	Myocardial infarction	MT	[42]
33	IONP	12	Exosome	Spinal cord injury	MT	[43]
34	IONP	12	Exosome	Ischemic stroke	MT	[44]
35	IONP	20–30	Exosome	Myocardial infarction	MT	[45]
36	Fe_3_O_4_	5	Exosome	Tumor	Photothermal therapy	[46]

–The relevant information was not found

A total of 36 literature studies are counted in Table 1. There are 14 articles on loading MNPs into cells, 2 articles on loading MNPs into vesicles, 11 articles on exosomes and 6 articles on liposomes. Table 1 shows that most researchers prefer to use MNPs of approximately 10 nm in the field of nanomedicine, because there are 25 literature studies introducing MNPs of 5–15 nm, accounting for 69%. This is consistent with the results in the literature [47, 48]. MNPs (< 200 nm) are rapidly filtered by the spleen and liver and cleared by the kidney at 2 nm, while 10-nm particles easily enter the circulatory system. Moreover, in the process of preparing MNPs, ultra-small magnetic particles (< 5 nm) have very good dispersion and stability in the organic coating. However, the response to external magnetic field is poor. Therefore, it is less used for magnetic targeting in cell therapy and more used as MRI contrast agent. When the size increases to 10 nm, it not only enhances the dispersion, but also enhances the response to the external magnetic field. In addition, with the continuous expansion of the scale, its agglomeration phenomenon is becoming more and more serious. Combined with the results summarized in the literature [6], the hydrodynamic size advantages [47] and optimization [49] of MNPs, we believe that a core diameter of approximately 10 nm has great advantages. At the same time, when preparing MNPs-labeled exosomes, the size of exosomes should be considered to facilitate the loading of MNPs. In addition, there are two main types of MNPs, namely Fe_3_O_4_ and γ-Fe_2_O_3_, but γ-Fe_2_O_3_ is better and safer for cells because ferric iron causes less damage to the recipient cells.

## Effect of the magnetic field on the cells labeled by MNPs

**Fig. 1:**
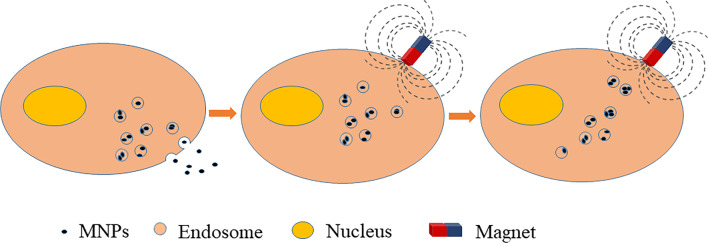
Changes of MNPs phagocytized by cells in magnetic field

The process of labeling cells with MNPs includes simple culture, transfection agents, magnetoelectric perforation and magnetoacoustic perforation [50]. The small volume and negative surface charge of MNPs are conducive to their nonspecific adsorption on the plasma membrane, thus triggering their endocytosis pathway in cells [51]. MNPs were ingested by cells and encapsulated in endosomes inside the cells. The endosomes were located close to the cytoplasm and away from the nucleus and not affected the normal activity of the cell. Analysis of the intracellular MNP distribution in the literature [17] showed that the MNP vesicles captured by cells diffuse along the cytoplasm and respond to magnetic fields [52], as shown in Fig. 1. A review [13] indicated that endosomes were elongated and aligned in the direction of the magnetic force line after the cells had phagocytized anionic colloidal ferromagnetic nanoparticles into the endosomes. In addition, the cells movement depended on the gradient in the magnetic field instead of feeling the strength [53–55]. The cells migrated toward the region with the maximum gradient. The study on the survival rate and proliferation rate of BMSCs under a high-intensity static magnetic field suggested that it had the least effect on cell proliferation within 24 h [56]. The effect depended on the degree of iron load [4]. If applied for a short time, cells can withstand a higher magnetic force. Moreover, stem cells were induced to differentiate by an extremely low-frequency magnetic field to obtain an ideal phenotype [57–59]. A study [51] revealed that the rotational movement of SPIONs induced by a dynamic magnetic field led to changes in cell membrane permeability and even apoptosis, because applying magnetic force to SPIONs distorted the inner membrane of the cell [13]. At the same time, the vesicles encapsulating MNPs not existed in cells for a long time and were soon excreted by cells to form free labeled extracellular vesicles.

**Fig. 2:**
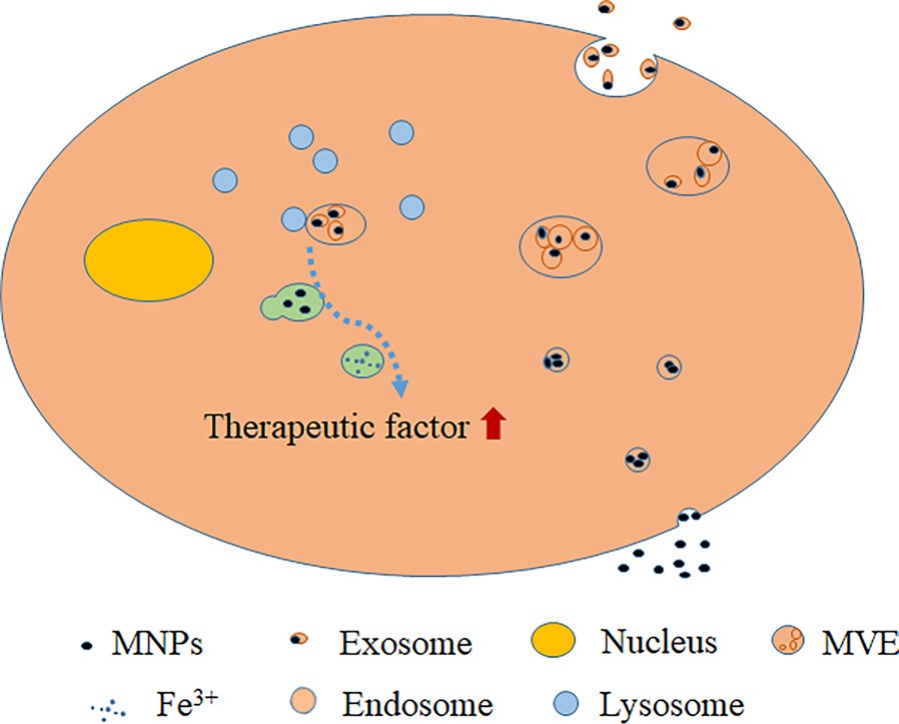
Cellular changes after phagocytosis of MNPs

In summary, the key is to pay attention to the load of MNPs, the strength and time in the magnetic field and the preparation time of cells' sample. Because of the long time, the vesicles encapsulating MNPs were produced in the test, as shown in Fig. 2.

## Application of MNP-labeled membrane systems in biomedicine

### Application of MNP-labeled cells in biomedicine

Stem cells and secreted vesicles have multidirectional differentiation potential, migrate to inflammatory sites and repair tissues and regenerate [60]. Therefore, they are widely used in cell therapy and have great prospects in the field of regenerative medicine. Stem cells have homing ability. However, they still have limitations and deficiencies. When stem cells are injected intravenously into the body, they are easily dispersed by blood flow and cleared by organisms, resulting in difficult localization, a short half-life and a low retention rate. To enhance targeting ability, prolong the retention rate and enhance the therapeutic effect of stem cells, MNPs are introduced. In addition, it was also important to minimize the injection dose, the toxicity and the side effects on healthy tissues and to cause the labeled cells or EVs to accumulate in the target tissue to reduce the in vitro administration concentration and frequency. As shown in Table 1, stem cells were labeled with MNPs and then injected into organisms under the magnetic field to treat lung injury [39], atrial fibrillation [61], coronary artery embolism [62], vascular diseases [63], systemic osteoporosis [36], retinitis [64], high intraocular pressure [30], Parkinson’s disease [40, 65], myocardial infarction [42] and spinal cord injury [43] and to regenerate articular cartilage and bone [66–71].

As shown in Fig. 2, MNP is engulfed by stem cells to form endosomes, which are then transformed into MVEs. The MVEs have two fates. One is to combine with lysosomes (low pH) in cells, be further digested and decomposed into Fe^3+^. The free metal ions released into the cytoplasm promote pathways of JNK activation and c-Jun phosphorylation, cause the upregulation of a variety of cytokines in cells, and induce angiogenesis, anti-apoptosis and anti-inflammatory [44]. Moreover, in the treatment of myocardial infarction, it was found to induce the increased expression of connexin 43 (Cx43) in H9c2 [42]. However, the other is excreted outside the cell in the form of extracellular vesicles. At the same time, exosomes prepared with MSC-IONP also contained a large number of therapeutic growth factors [44].

### Application of MNP-labeled EVs in biomedicine

In the past decade, there have been an increasing number of studies on EVs, especially exosomes (50–150 nm). Due to the biological characteristics such as low immunogenicity and drug delivery, they have better application prospects in the field of tissue regeneration [6]. EVs are effectors of intercellular communication and act as natural endogenous carriers. To improve their low separation rate and insufficient targeting ability, MNPs and magnetic localization were used to enhance the directional distribution ability of EVs. To internalize SPIONs directly into EVs, electroporation, natural incubation and other methods were used. However, the membrane is incomplete due to electroporation. MNP-labeled cells' secretory vesicles are the primary choice for natural processes. Under the external magnetic field, MNP-labeled EVs were concentrated and enriched at the target position for tissue repair and regeneration. Many studies have used this method to improve and treat scar formation in acute and chronic porcine myocardial infarction [72], ischemic stroke [44], skin trauma [27], spinal cord injury [43, 73], infarcted heart [22], wound healing [23], bone and angiogenesis [74], and myocardial infarction [45].

### Application of magnetic liposomes in biomedicine

Liposomes are similar to EVs released by cells. Synthetic liposomes are usually spherical closed structures composed of lipid bilayers enclosing the internal hydrophilic chamber [75]. The size of liposomes ranges from 20 nm to several microns [76]. Like vesicles released by cells, liposomes can encapsulate molecules with different solubilities. Liposomes have the advantage of encapsulating MNPs without being affected by enzyme degradation, but there are no proteins or other biomolecules from precursor cells [16]. At the same time, loading hydrophilic USPIO into the hydrophilic chamber of liposome can also achieve the magnetic targeting [77], drug delivery and MRI imaging [21, 78, 79] and generate multifunctional liposomes [77, 80]. Moreover, the preparation of liposomes is simple and controllable, so researchers have given increasing attention to the use of liposomes. Under a permanent magnetic field, magnetic liposomes deform into slender ellipsoids [81]. A study [82] first reported the fusion of dry magnetic liposomes and cell membrane models, evaluated their interactions (giant monolayer vesicles, GUVs) and considered the future application in drug delivery of such magnetic systems. Ideally, the target release system allows external control of the time and dose of products released at the target location [83].

In summary, the application of the MNP-labeled membrane systems has treated many diseases successfully. In addition, the method can also be extended to the cell localization of other organs and provide a useful tool for systematic cell therapy. At the same time, it can also treat acute organ failure with high mortality. For example, acute lung injury and acute kidney injury are caused by COVID-19 or other diseases. However, there are few studies on these diseases. Combined with the rapid targeting enrichment and the therapeutic advantages of stem cells and exosomes, it will be possible to treat acute organ failure.

## Tumor treatment with magnetic hyperthermia

In addition to tissue repair and regeneration, the combination of MNPs and membrane systems is also used to treat tumors. By changing the frequency of the external magnetic field, the high-speed movement of MNPs can be controlled. The magnetic fields mentioned here include the high-frequency and the low-frequency magnetic field. The high-frequency AMF makes MNPs in organism rotate at high speed, increase membrane permeability [11, 16, 21], enhance drug release, and even tear cell membrane [83]. It can also convert magnetic energy into heat to kill tumor cells. Protect the surrounding environment while the local temperature far exceeds the body temperature [12]. The damaging effect of magnetic hyperthermia on microglial BV2 cells [17] and increasing drug release [84] were studied. However, under a low-frequency magnetic field, the mechanism of destroying the membrane was mechanical and not dependent on heat. Therefore, nonspecific heating of surrounding tissues caused by AMF is avoided [83].

According to the literature for magnetic hyperthermia, in this paper, we mainly reviewed the combination of MNPs and membrane systems for magnetic hyperthermia to treat tumors. Therefore, the use of only heated MNP to kill tumors is not described here. Cell communication promotes tumor development through vesicles. It is believed that the vesicles are used as Trojan horses to provide a therapeutic payload for cancer cells [76]. Therefore, exosomes in blood were combined with MNPs to target cancer [26]. Recently, it was reported that SPION-modified exosomes transferred TNF-α to cancer cells through magnetic targeting and significantly inhibited tumor growth [28].

With regard to the study of magnetic liposomes, the literature studies [81, 85, 86] have reported the possibility of using magnetic liposomes and in vitro magnets to treat solid tumors. Some reports [84, 87] prepared multifunctional magnetoliposomes loaded with the anticancer drug DOX to inhibit cancer. In high-frequency magnetic field, multifunctional liposomes cause perforation effect or change the permeability of vesicle membrane through local heating to increase drug release [88]. This method opens up a new prospect for the development of intelligent drug delivery system.

## MRI application of MNP-labeled membrane systems

MNP-labeled membrane systems were used for three-dimensional noninvasive imaging positioning to achieve real-time monitoring of biological distribution in vivo [47, 89–92], as well as MRI of embryonic tissue [93, 94], adipose [95, 96], human adipose-derived stem cells [97, 98] and extracellular vesicles [99–101]. The dual exogenous substances in which cells are incubated with photosensitizers and MNPs [76] provide magnetic and optical responsiveness to vesicles for treatment and monitoring distribution. In addition to the situation described above, MNPs were encapsulated in liposomes together with fluorescent nanoparticles [102], rhodamine labeling [103] and quantum dots [104] for tracking. In conclusion, the application of MNP-labeled membrane systems can simultaneously have the advantages of treatment and visualization, so as to timely monitor the embolism caused by foreign substances and evaluate the metabolism.

## Conclusions and expectations

This paper reviews the research progress of the application of MNP-labeled membrane systems in biomedicine. They have a wide range of applications in medicine and open up a potential application prospect for the directional positioning of biological entities. Through the perfect combination and utilization of magnetic targeting, MRI and magnetic hyperthermia, many diseases are expected to be successfully treated. In particular, through the continuous progress of magnetic field design, noninvasive treatment of deep diseases can be realized, not just on the surface of the body. The development of MNPs in precision medicine needs more exploration. In the future, it is expected to achieve more mature, comprehensive (systematic) and accurate (positioning) disease treatment in medicine.

## Data Availability

The data used to support the findings of this study are available from the corresponding author upon request.

## Abbreviations

SPIONs: Superparamagnetic iron oxide nanoparticles
MNPs: Magnetic nanoparticles
USPIO: Ultra-small superparamagnetic iron oxide
MT: Magnetic targeting
ADSCs: Human primary adipose-derived stem cells
hMSCs: Human mesenchymal stem cells
MSCs: Mesenchymal stromal cells
NSCs: Neural stem cells
ESCs: Embryonic stem cells
EVs: Extracellular vesicles
BBB: Blood–brain barrier
EPCs: Endothelial progenitor cells
DOX: Doxorubicin
MRI: Magnetic resonance imaging
TNF-α: Tumor necrosis factor
IONP: Iron oxide nanoparticle
H9C2: Cardiomyoblasts
Cx43: Connexin 43
MVEs: Mature multivesicular endosomes Alternating magnetic field(AMF)
AMF: Alternating magnetic field
